# Addiction of primary cutaneous **γδ** T cell lymphomas to JAK/STAT signaling

**DOI:** 10.1172/JCI180417

**Published:** 2025-04-15

**Authors:** Yue Zhang, Julia A. Yescas, Kristy Tefft, Spencer Ng, Kevin Qiu, Erica B. Wang, Shifa Akhtar, Addie Walker, Macartney Welborn, Martin Zaiac, Joan Guitart, Aamir M. Qureshi, Youn H. Kim, Michael S. Khodadoust, Naiem T. Issa, Jaehyuk Choi

**Affiliations:** 1 Department of Dermatology, Northwestern University, Chicago, Illinois, USA.; 2 Department of Dermatology, Indiana University, Indianapolis, Indiana, USA.; 3 Division of Dermatology, Department of Medicine, and Department of Pathology and Immunology, Washington University School of Medicine, St. Louis, Missouri, USA.; 4 Department of Dermatology, Stanford University, Palo Alto, California, USA.; 5 Department of Dermatology, Cleveland Clinic, Cleveland, OH, USA.; 6 Department of Dermatology, University of Florida, Gainesville, Florida, USA.; 7 Department of Dermatology, Florida International University, Miami, Florida, USA.; 8 Incyte Corporation, Wilmington, Delaware, USA.; 9 Division of Oncology, Stanford Medicine, Stanford, California, USA.

**Keywords:** Dermatology, Oncology, Bioinformatics, Cancer, Skin cancer

To the Editor: Primary cutaneous γδ T cell lymphoma (PCGDTL) is an aggressive malignancy with a median survival of just 31 months and no effective standard therapies (1). These lymphomas frequently harbor mutations in the JAK/STAT signaling pathway (2). We hypothesized that single-agent JAK inhibition could provide therapeutic benefit in PCGDTLs with actionable JAK/STAT pathway mutations. Here, we report near-complete responses in 2 patients treated with ruxolitinib or cerdulatinib. However, both patients ultimately experienced disease relapse, driven by acquired mutations in *JAK1*, *JAK3*, or *STAT5B*.

Patient 1, a 90-year-old female, presented with ulcerated facial plaques that rapidly progressed to tumors, spreading to the neck, trunk, and extremities (Figure 1A, Supplemental Table 1, and Supplemental Figure 1, A–D; supplemental material available online with this article; https://doi.org/10.1172/JCI180417DS1). A biopsy revealed an atypical dermal lymphocytic infiltrate positive for T cell receptor δ (TCR-δ), with tumor, lymph node, metastasis (TNM) staging indicating T_3B_N_0_M_0_ disease (Supplemental Table 1 and Supplemental Figure 1, E–N). Whole-genome sequencing (WGS) identified a *SOCS1* deletion, a key regulator of JAK/STAT signaling, and clonotype analysis showed a Vδ1Vγ5 rearrangement (Supplemental Table 1, Figure 1B, and Supplemental Figure 2, A and B). RNA-Seq and gene set enrichment analysis (GSEA) confirmed upregulation of the JAK/STAT pathway compared with nonactivated Vδ1 T cells, with positive phosphorylated STAT3 (pSTAT3) on immunohistochemistry (Figure 1C, Supplemental Figure 1M, and Supplemental Figure 2C).

Unable to tolerate chemotherapy, the patient was treated with the JAK1/2 inhibitor ruxolitinib. After 4 weeks, she showed near-complete regression, with a 63% reduction in skin disease burden (modified severity-weighted assessment tool [mSWAT] 16 to 6), significant tumor shrinkage, and clearance of plaques (Figure 1A and Supplemental Figure 1, A–D). However, at 8 weeks, the disease progressed rapidly, and new plaques emerged. Histopathology confirmed recurrence with the same Vδ1Vγ5 clone (Supplemental Figure 2B). Ultimately, she was hospitalized for and died of sepsis. WGS of relapsed disease revealed a newly acquired STAT5B p.N642H mutation, which was sufficient to induce resistance to ruxolitinib (Figure 1, D and E, Supplemental Figure 1N, Supplemental Figure 2A, and Supplemental Figure 4, A–D).

Patient 2, a 74-year-old female, presented with a 1-year history of enlarging red-to-violaceous plaques/nodules on the lower legs and inguinal lymphadenopathy (Figure 1F and Supplemental Figure 3, A and B). Histology confirmed PCGDTL, showing infiltration by large, atypical lymphocytes positive for TCR-δ with a clonal Vδ1Vγ5 chain; staging indicated T_2C_N_1_M_0_ disease (Supplemental Table 1, Supplemental Figure 2D, and Supplemental Figure 3E). Following disease progression after 1 year of pralatrexate and brentuximab, next-generation sequencing (NGS) revealed a JAK3 p.A573V mutation (Figure 1G and Supplemental Figure 2A). JAK/STAT pathway activation was confirmed in JAK inhibition–naive biopsies (Figure 1H and Supplemental Figure 2E).

The patient enrolled in a phase II trial of the dual SYK and JAK inhibitor cerdulatinib (3). By 7 weeks, the patient had an 86% reduction in skin disease burden (mSWAT 20 to 2.9) (Figure 1F and Supplemental Figure 3, B and C). Treatment was limited by grade 3 adverse events. Dosing fluctuated below the target level. At 16 weeks, new biopsy-confirmed PCGDTL tumors emerged (Supplemental Figure 3D). Heme-STAMP (Stanford tumor actionable mutation panel for hematopoietic and lymphoid neoplasms) detected JAK3 p.A573V in peripheral blood, indicating leukemic progression, with a peak WBC count of 101 k/μL (Supplemental Figure 2A). A bone marrow biopsy confirmed 90%–100% involvement of PCGDTL. The patient deteriorated rapidly and died. NGS identified a new JAK3 p.M511I mutation in the recurrent skin tumor and new JAK1 p.L783F and subclonal JAK1 p.T901A mutations in the blood (Figure 1, G and I, and Supplemental Figure 2A). Functional studies showed that JAK3 p.M511I and JAK1 p.L783F, but not JAK1 p.T901A, promoted transformation in Ba/F3 cells (Supplemental Figure 4, E and F). Moreover, the combination of genetic variants seen in the relapsed tumor were sufficient to induce resistance to cerdulatinib (Figure 1J).

Here, we demonstrate significant short-term responses to JAK inhibitors in Vδ1 PCGDTLs. To contextualize our findings, we analyzed data from a recent phase IIb trial of ruxolitinib in T cell lymphomas, which reported an overall response rate of 25% for γδ-TCLs (4). Notably, 3 of the 4 γδ-TCL patients in the trial harbored the STAT5B p.N642H mutation, but only 1 achieved a partial response lasting under 6 months. Combined with our results, this suggests that *STAT5B* mutations may contribute to both primary and acquired resistance to JAK inhibition.

Our findings highlight the idea that a subset of PCGDTLs are addicted to JAK inhibitors. Acquired resistance to JAK inhibitors occurs through pathway reactivation. In this way, PCGDTLs resemble melanomas that are addicted to MAPK signaling (5). Analogous to melanoma, newer treatments may require combination regimens that inhibit multiple components of the JAK/STAT pathway to forestall resistance.

## Supplementary Material

Supplemental data

Supplemental table 1

Supporting data values

## Figures and Tables

**Figure 1:**
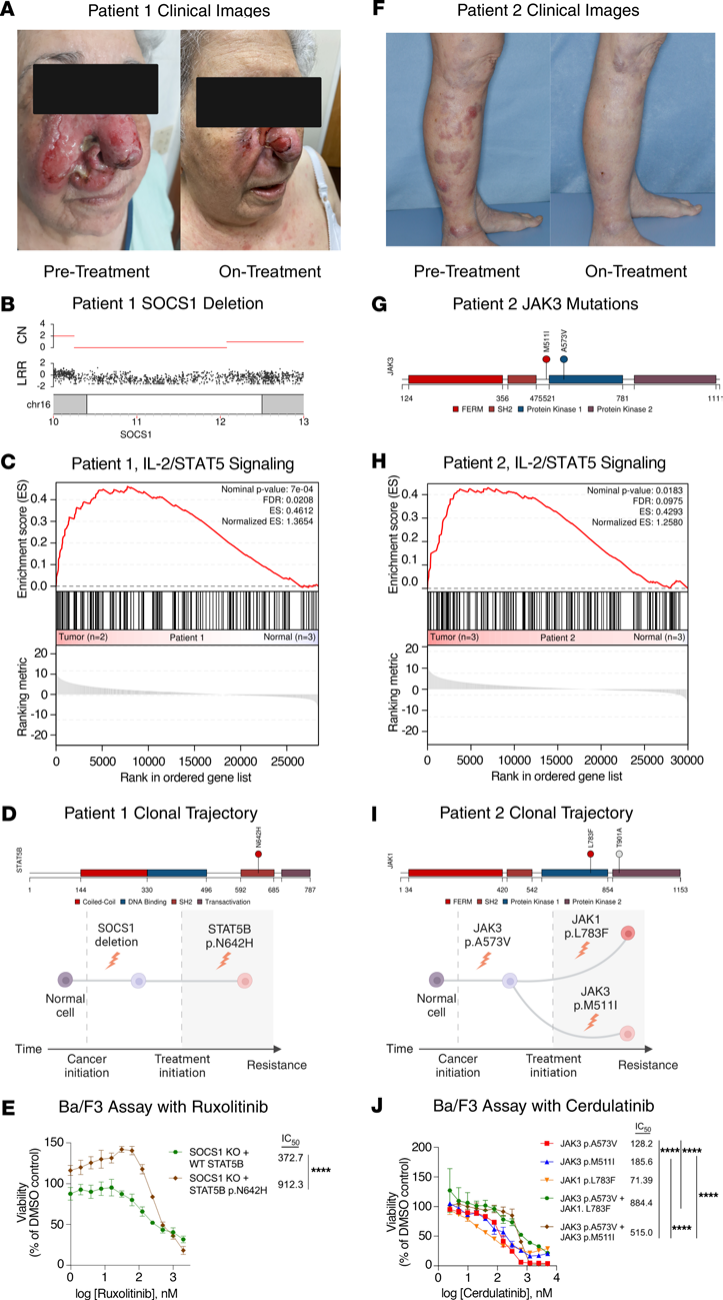
PCGDTL regression with JAK inhibitor monotherapy and mechanisms of acquired resistance. (**A**) Clinical response of patient 1 to ruxolitinib. (**B**) WGS of PCGDTL in patient 1 revealed a deletion in p13.13, containing the *SOCS1* locus. CN, copy number; LRR, log R ratio. (**C**) GSEA of Hallmark IL-2/STAT5 signaling of 2 tumor samples against 3 normal mature Vδ1 T cells revealed increased activity. (**D**) The post-relapse PCGDTL contained an acquired STAT5B p.N642H mutation. (**E**) STAT5B p.N642H increased the IC_50_ of ruxolitinib in *SOCS1*-deficient Ba/F3 cells. (**F**) Clinical response of patient 2 to cerdulatinib. (**G**) NGS of PCGDTL in patient 2 revealed a JAK3 p.A573V mutation and a post-relapse JAK3 p.M511I mutation. (**H**) GSEA of Hallmark IL-2/STAT5 signaling of 3 tumor samples against 3 normal mature Vδ1 T cells revealed increased activity. (**I**) Post-relapse leukemic PCGDTL contained an additional JAK1 p.L783F and subclonal JAK1 p.T901A mutations. (**J**) JAK3 p.M511I and JAK1 p.L783F increased the IC_50_ of cerdulatinib in Ba/F3 cells containing JAK3 p.A573V in the absence of IL-3. ****P < 0.0001, by multiple-comparison 2-way ANOVA (**E** and **J**). Created with BioRender.com.

## Version 1 04/15/2025

Electronic publication
