# Supplementary material for: Addiction of primary cutaneous γδ T cell lymphomas to JAK/STAT signaling
Source: J Clin Invest. 2025 Apr 15;135(8):e180417. doi: 10.1172/JCI180417 (PMC11996904; doi:10.1172/JCI180417)
Supplement: Supplemental data [file jci-135-180417-s151.pdf]

Supplementary Figures and Methods

This appendix has been provided by the authors to give readers additional information about the work.

Supplementary Figures and Methods to:

Addiction of primary cutaneous gamma-delta T-cell lymphomas to JAK-STAT signaling

Yue Zhang[*1,2], Julia A. Yescas[*1], Kristy Tefft[*1], Spencer Ng[**1,3], Kevin Qiu[1], Erica B. Wang[4], Shifa Akhtar[5], Addie Walker[6], Macartney Welborn[6], Martin Zaiac[7], Joan Guitart[1], Aamir Qureshi[8], Youn H. Kim[4], Michael S. Khodadoust[4,9], Naiem T. Issa[**5], Jaehyuk Choi[#1]

[1] Department of Dermatology, Northwestern University, Chicago, IL.
[2] Department of Dermatology, Indiana University, Indianapolis, IN.
[3] Department of Dermatology, Medicine, and Immunology, Washington University School of Medicine, St. Louis, MO.
[4] Department of Dermatology, Stanford University, Palo Alto, CA.
[5] Department of Dermatology, University of Miami, Miami, FL.
[6] Department of Dermatology, University of Florida, Gainesville, FL.
[7] Department of Dermatology, Florida International University, Miami, FL.
[8] Incyte Corporation, Wilmington, DE.
[9] Division of Oncology, Stanford Medicine, Stanford, CA.

*Co-first authors by agreement
**Equal contribution
#Corresponding author, 303 E Superior, Room 5-115, Chicago, IL 60611,
jaehyuk.choi@northwestern.edu, (312)-503-1134

DISCLOSURES

J.G. received consultation fees from Kyowa Kirin and grants from Elorac
A.Q. has affiliations with Galderma, Incyte, Sun Pharma, Ortho Dermatologics, Valeant Pharmaceuticals.
Y.H.K. received research funding from Kyowa Kirin, Drenbio, Corvus, and Innate.
M.S.K. received research funding from CRISPR Therapeutics.
J.C. received consultation fees from Jansen and is a stakeholder/co-founder of Moonlight Bio.

Table of Contents Page 3

Acknowledgements	Page 4
Data Availability	Page 5
Methods	Page 6
Supplemental Figure 1	Page 11
Supplemental Figure 2	Page 13
Supplemental Figure 3	Page 15
Supplemental Figure 4	Page 16
References	Page 17

Acknowledgements

We thank the patients and their families who contributed to this study. We thank the Northwestern Skin Disease Research Center, the Robert H. Lurie Comprehensive Cancer Center, the Skin Biology and Diseases Resource-Based Center, and the Northwestern University Research Computing Services for their assistance. We thank Admera Health for performing next generation sequencing. This study was supported in part by the National Institutes of Health (J.C., grant 5R01-CA260064-03), the Lymphoma and Leukemia Society Scholar Award (1377-21), and the Bakewell Foundation. The study was supported in part by the American Skin Association (Y.Z., David Mandelbaum Medical Student Grant). The study was supported by the American Society of Hematology (Y.Z., Hematology Opportunities for the Next Generation of Research Scientists). We thank the Northwestern Dermatology Clinical Trials Unit for their support (Y.Z., K.T.). This research was supported in part by resources provided by the Northwestern University Skin Biology and Diseases Resource-based Center (P30 AR075049), Chicago, IL with support from the NIH/NIAMS. Any opinions, findings, and conclusions or recommendations expressed in this material are those of the author(s) and do not necessarily reflect the views of the Northwestern University Skin Biology and Diseases Resource-based Center or the NIH/NIAMS. Figures created with BioRender.com. Jakafi (ruxolitinib) is produced by Incyte. Cerdulatinib is produced by Alexion.

Data availability

Original sequencing files of patient samples with appropriate IRB approval are available at the dbGAP accession phs003837.v1.p1. Data used to create the figures of this manuscript are available in the supporting data values file.

Methods

Sex as a biological variable

Both patients in this study were female. Although our cohort is limited in size (n=2), we did not find evidence that sex influenced the results.

Diagnostic criteria of primary cutaneous gamma-delta T-cell lymphoma

Patients met accepted criteria of PCGDTL (1, 2). Both patients had primary skin involvement without extracutaneous disease at the initial presentation and clinicopathologic correlation was consistent with epidermotropic gamma-delta T-cell lymphoma. A biopsy of a representative lesion from each patient was interpreted by at least one expert pathologist (AW, JG). The infiltrating malignant lymphocytes were determined to be γδ-rearranged by positive IHC staining for TCR-δ, and TCR sequencing using MiXCR confirmed the presence of a clonal and productive γδ-TCR (3).

Patient disease assessment and modified Severity Weighted Assessment Tool

The patient staging was performed in accordance with Olsen et al. 2022(1) for non-mycosis fungoides/Sezary syndrome. The staging was reviewed by the authors of the manuscript for accuracy. Patient disease burden was recorded using the modified Severity Weighted Assessment Tool (mSWAT) as described in Olsen et al. 2022 for non-mycosis fungoides/Sezary syndrome. Tumor biopsies were reviewed by multiple pathologists and a diagnosis of epidermotropic PCGDTL was made based on histologic pattern and staining for TCR-δ.

HemeSTAMP

The Stanford Actionable Mutation Panel for Hematopoietic and Lymphoid Neoplasms (HemeSTAMP) is a Clinical Laboratory Improvement Amendments (CLIA)-certified targeted next-generation sequencing panel. It detects single nucleotide variants, short insertion-deletions, and selected gene fusions in 164 genes recurrently altered in myeloid and lymphoid neoplasms. The genomic features are interrogated to achieve a minimum analytic detection limit of 5% for SNVs and insertion-deletion variants. The panel detects known drivers of PCGDTL, including but not limited to JAK/STAT pathway members *JAK1*, *JAK2*, *JAK3*, *SOCS1*, STAT1, *STAT3*, *STAT5B*, and *STAT6*; and MAPK pathway members *AKT1*, *BRAF*,

HRAS, *KRAS*, *MAPK1*, *MTOR*, *MYC*, *NF1*, *NRAS*, *PIK3CA*, *PIK3CD*, and *PTEN*. HemeSTAMP has been previously used by authors in studies investigating CCR4 mutations and PD-L1 structural variants in cutaneous T-cell lymphoma(4, 5).

High-throughput short-read sequencing

DNA and RNA were extracted from formalin-fixed, paraffin-embedded (FFPE) samples. Whole genome sequencing was performed by Admera (South Plainfield, NJ) using the Kapa Hyper Prep (Roche) kit. Samples were sequenced to a target of 600M paired-end reads (300M reads on each side) of at least 150 BP. RNA-sequencing was performed by Admera (South Plainfield, NJ) using the KAPA RNA HyperPrep Kits with RiboErase (HMR) (Roche) kit. Samples were sequenced to a target of 60M paired-end reads (30M reads on each side) of at least 150 BP.

Whole genome sequencing analysis

Raw paired-end fastq reads from whole genome sequencing were aligned to GRCh38.p13 with BWA (6) and standardized with base quality score recalibration, indel realignment, and duplicate removal using GATK 4.4.0.0 best practices, as previously described (7-10). SNP and indel discovery was performed using MuTect2.0 and applying a contamination filter, removal of strand orientation artifacts, and sequencing artifacts (11). We removed artifactual mutations resulting from FFPE fixation using SOBDetector (12). An internal normal panel was used to denoise contaminating germline variants, as previously described (13-17). Variants occurring frequently in the gnomAD 3.0 database in healthy populations were removed (18). We looked for single-nucleotide drivers by either identifying missense mutations found recurrently in the COSMIC somatic mutation database and our internal database of PCGDTL and T-cell lymphoma (TCL) mutation calls or damaging mutations in putative tumor suppressors described previously (19). Lollipop plots of individual SNVs were generated using the trackViewer Bioconductor package (20). Copy-number variants were called using ASCAT (21) and with GATK4CNV trained on an in-house panel of normal. SNPs were assessed at Affymetrix 6.0 sites and heterozygous SNPs were chosen in downstream processing of segmentation. Segmental duplications and regions of poor mapping score were removed and copy number regions with greater than 25% overlap

with germline variants in gnomAD were removed. The sum of allele-specific copy number, raw LRR, and raw BAF were plotted along chromosomal bands and coordinates using karyoploteR (22).

RNA-sequencing analysis

Raw paired-end RNA-sequencing FASTQ reads were trimmed using CutAdapt 4.1(23) and were aligned to GRCh38.p13 with GENCODE v30 transcripts using STAR v.2.7.10 (24), and counts were generated using HTSeq 2.0.2 (25). Gene expression values used in heatmaps were transformed using variance stabilizing transformations (26, 27). Gene expression data was compared to publicly available RNA sequencing data from mature Vδ1 gamma-delta T-cells from healthy adults age >18 (GSE128163), specifically SRR8713468, SRR8713469, and SRR8713468(28). Differentially expressed genes between tumor and normal samples were called using DESeq2 1.43.1 (29). Heatmaps were generated using ComplexHeatmap (30) R package row-normalized variance stabilizing transformed gene expression values (30). Gene set enrichment plots, normalized enrichment scores, and p-values were generated using GSEAPreranked 4.3.2 (31, 32) to assess STAT3 and STAT5 target gene expression as defined by the Hallmark IL2-STAT5 Signaling and Hallmark IL6-JAK-STAT3 Signaling gene sets from MSigDB(33). T-cell receptor clonotyping was performed using MiXCR as previously described (3).

Immunohistochemistry

TCR delta H41 (Santa Cruz Biotechnologies) and pSTAT3 (Cell Signaling Technologies, Clone D3A7) were incubated for 4 hours in 1:100 dilution before secondary antibody incubation and subsequent counterstaining with hematoxylin and eosin.

Cell line generation and culture

JAK1, JAK3, and STAT5B cDNA were purchased from Twist Bioscience. The cDNA was mutagenized by Gibson cloning and subcloned into pCDH-CMV-MCS-EF1-copGFP vector or pCDH-CMV-MCS-EF1-copRFP (Systems Biosciences). To knock out SOCS1, guide RNA specific to mouse SOCS1 (top sg: CACCGGGACGCCTGCGGCTTCTATT, bottom sg: AAACAATAGAAGCCGCAGGCGTCCC) were cloned into lentiviral expression vector (LentiCRISPRv2-mCherry) (#99154, Addgene).

HEK293T cells and Ba/F3 cells were maintained in Dulbecco`s modified eagle medium and RPMI-1640, respectively. Media were supplemented with 5% fetal bovine serum, penicillin/streptomycin (Sigma-Aldrich), and 1 mM HEPES (Gibco). Lentiviral particles were harvested from HEK293T and concentrated using Lenti-X Concentrator (Takara). Ba/F3 cells were transduced with concentrated virus and lentivirally transduced cells were selected by green fluorescent protein (GFP) and/or red fluorescent protein (RFP) expression.

BAF3 IL-3 withdrawal

Ba/F3 cells were maintained at a cell density of 0.25M/ml in the presence of 10 ng/ml IL-3 (Peprotech). Successfully transduced Ba/F3 cells were seeded at a cell density of 1M/ml in a 96-well plate in the presence or absence of IL-3 on day 0. Cell counts were obtained by flow cytometry.

Cell viability assay

Ba/F3 cells were cultured with Ruxolitinib (INCB018424, Selleckchem), Cerdulatinib (PRT062070, Selleckchem) or DMSO in a 96-well plate at a cell density of 1M/ml. Cell-titer Glo luminescent reagent (Promega) was added after 72 hours. Luminescence was measured using Biotek Cytation imaging reader. IC50 values were determined using a nonlinear regression model in GraphPad Prism version 10 (GraphPad Software).

Study approval and study oversight

Patient 1: Emergency Use Authorization (EUA) from the U.S. Food and Drug Administration (FDA) was obtained to utilize ruxolitinib to treat a single elderly adult patient with PCGDTL. This was done in cooperation with Incyte Corporation, which provided our study the medication and regulatory support. The study was approved by the Institutional Review Board of Mount Sinai Medical Center in Miami, Florida, USA. The patient was provided with written informed consent for treatment. Written informed consent was received for the use of the photographs and that the record of informed consent has been retained.

Patient 2: The patient was provided with written informed consent and was enrolled in a clinical trial evaluating the safety of cerdulatinib in relapsed/refractory T-cell non-Hodgkin lymphoma (Study ID: 13-

601, NCT 01994382)(34). This was performed in cooperation with Alexion Pharmaceuticals, Inc., who provided medication and regulatory support. This study was approved by the Institutional Review Board at Stanford University, Stanford, CA. Written informed consent was received for the use of the photographs and that the record of informed consent has been retained.

Statistics

Statistical differences among Ba/F3 groups were tested using multiple comparison 2-way ANOVA. GSEA p-value and FDR are calculated as described previously (31).

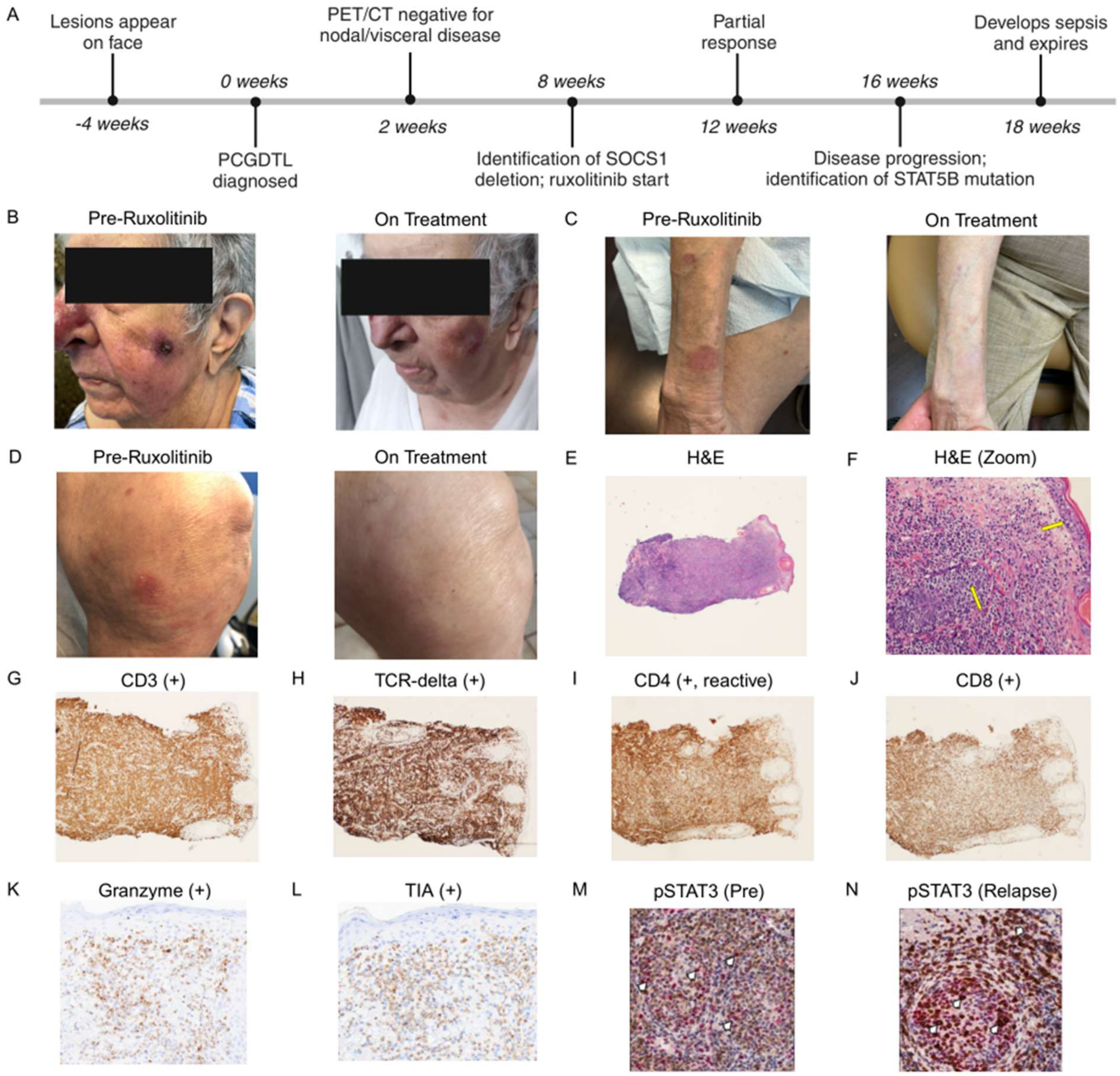

Supplemental Figure 1. Timeline, additional clinical photos, and histologic images, patient 1. (**A**) Clinical course of patient 1. (**B**) Regression of tumors on left face. (**C**) Regression of plaques on right forearm. (**D**) Regression of plaques on right knee. (**E**) Hematoxylin and eosin (H&E) stain at 2x and (**F**) 20x magnification (Zoom). Atypical lymphocytes exhibiting epidermotropism and adnexotropism highlighted by yellow arrows Immunohistochemistry at 4x revealing staining patterns for (**G**) CD3, (**H**) TCR-delta, (**I**) CD4, (**J**) CD8, (**K**) granzyme, and (**L**) TIA. In summary, the infiltrate was positive for CD3, CD8, granzyme B, pSTAT3, TIA, and TCR-delta, and negative for CD10, CD20, CD30, CD56,

BCL-2, and EBER (not displayed). (**M**) pSTAT3 (pre-treatment), and (**N**) pSTAT3 (post-relapse). The specimens display TCRδ (brown chromogen) and phospho-STAT3 (red chromogen), with co-expression of phosphorylated STAT3 in TCRδ positive cells. Created with BioRender.com.

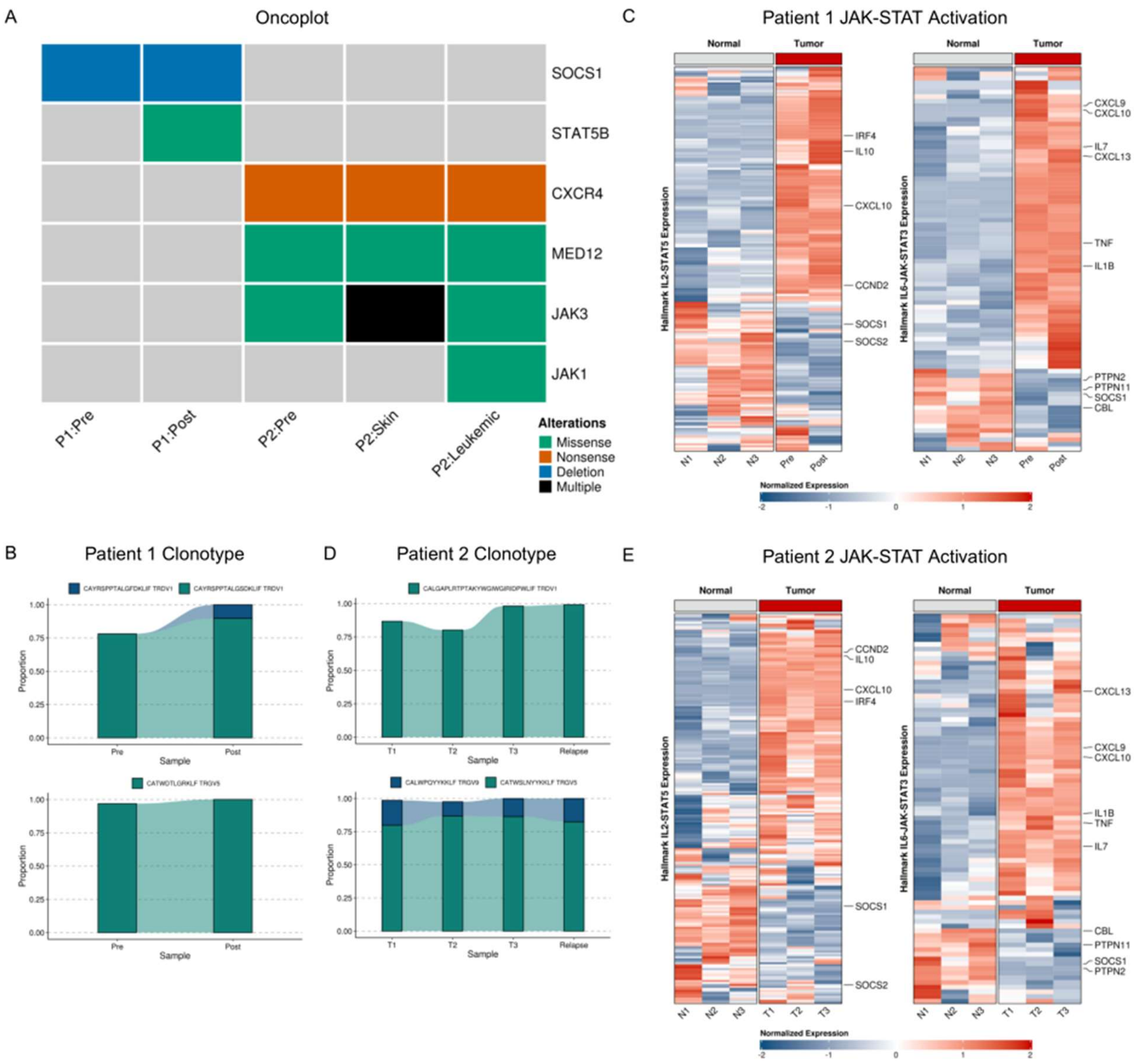

Supplemental Figure 2. Oncoplot, TCR clonality, and JAK-STAT activation. (**A**) Oncoplot of genes pre- and post-relapse of patient 1 and patient 2. (**B**) T-cell receptor clonality at two timepoints for patient 1. (**C**) JAK-STAT pathway activity for patient 1. Expression of effector cytokines *IL1B, IL7, IL10, TNF, CXCL9, CXCL10, CXCL13*; transcription factor *IRF4*; and cyclin *CCND2* were higher compared to normal Vδ1 cells. Expression of inhibitory genes *SOCS1, SOCS2, PTPN2, PTPN11, CBL* were lower compared to normal Vδ1 cells. (**D**) T-cell receptor clonality at four timepoints for patient 2. (**E**) JAK-STAT pathway activity for patient 2. Expression of effector cytokines *IL1B, IL7, IL10, TNF, CXCL9, CXCL10, CXCL13*; transcription factor *IRF4*; and cyclin *CCND2* were higher compared to normal Vδ1

cells. Expression of inhibitory genes *SOCS1, SOCS2, PTPN2, PTPN11, CBL* were lower compared to normal Vδ1 cells. Created with BioRender.com.

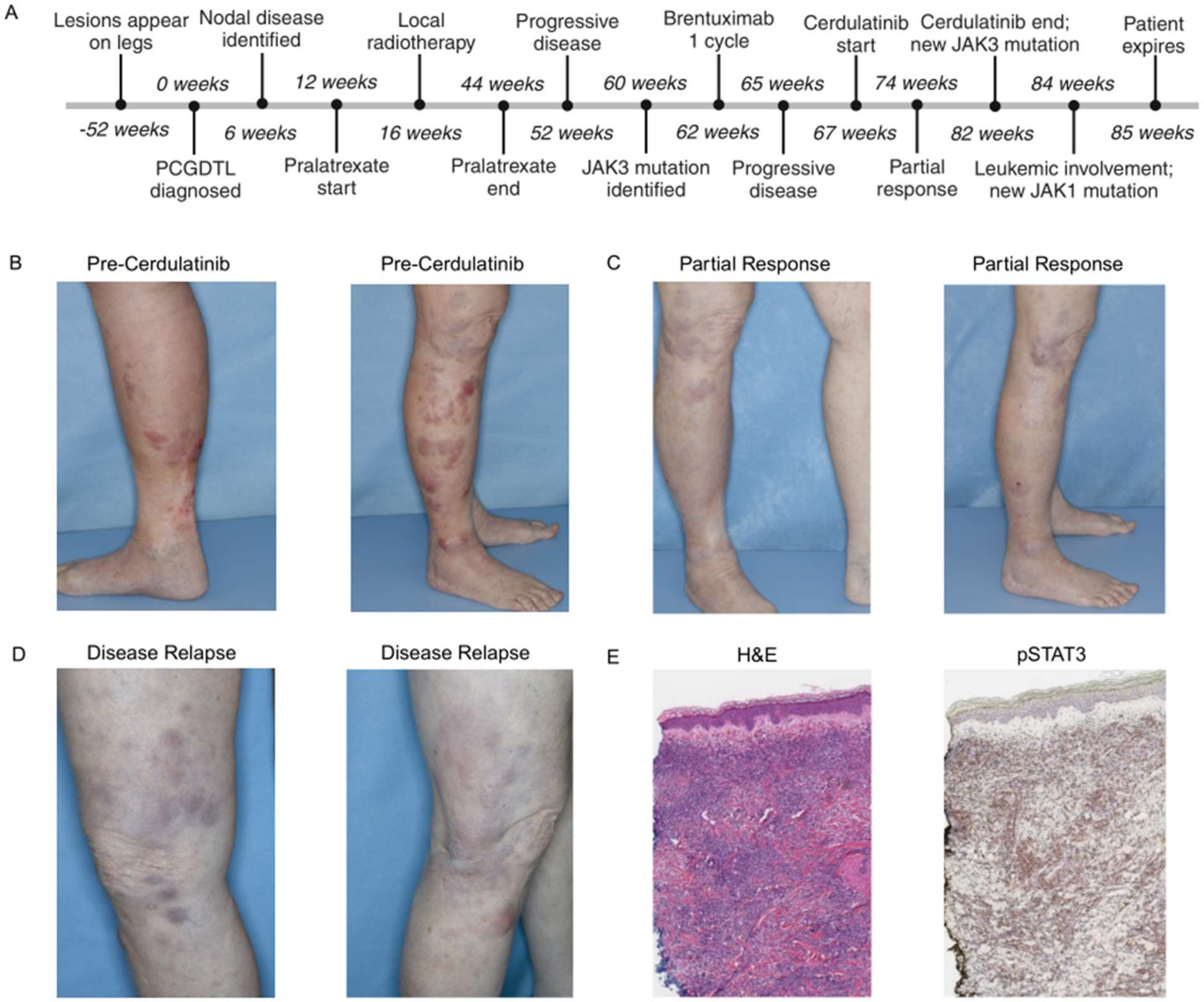

Supplemental Figure 3. Timeline and additional clinical photos, patient 2. (**A**) Clinical course of patient 2. (**B**) Leg lesions prior to cerdulatinib therapy. (**C**) Leg lesions after cycle 3 of cerdulatinib therapy, showing partial response. (**D**) Leg lesions after cycle 5 of cerdulatinib therapy, showing disease relapse. (**E**) Hematoxylin and eosin (H&E) stain at 20x magnification and pSTAT3 with brown chromogen at 20x magnification. Created with BioRender.com.

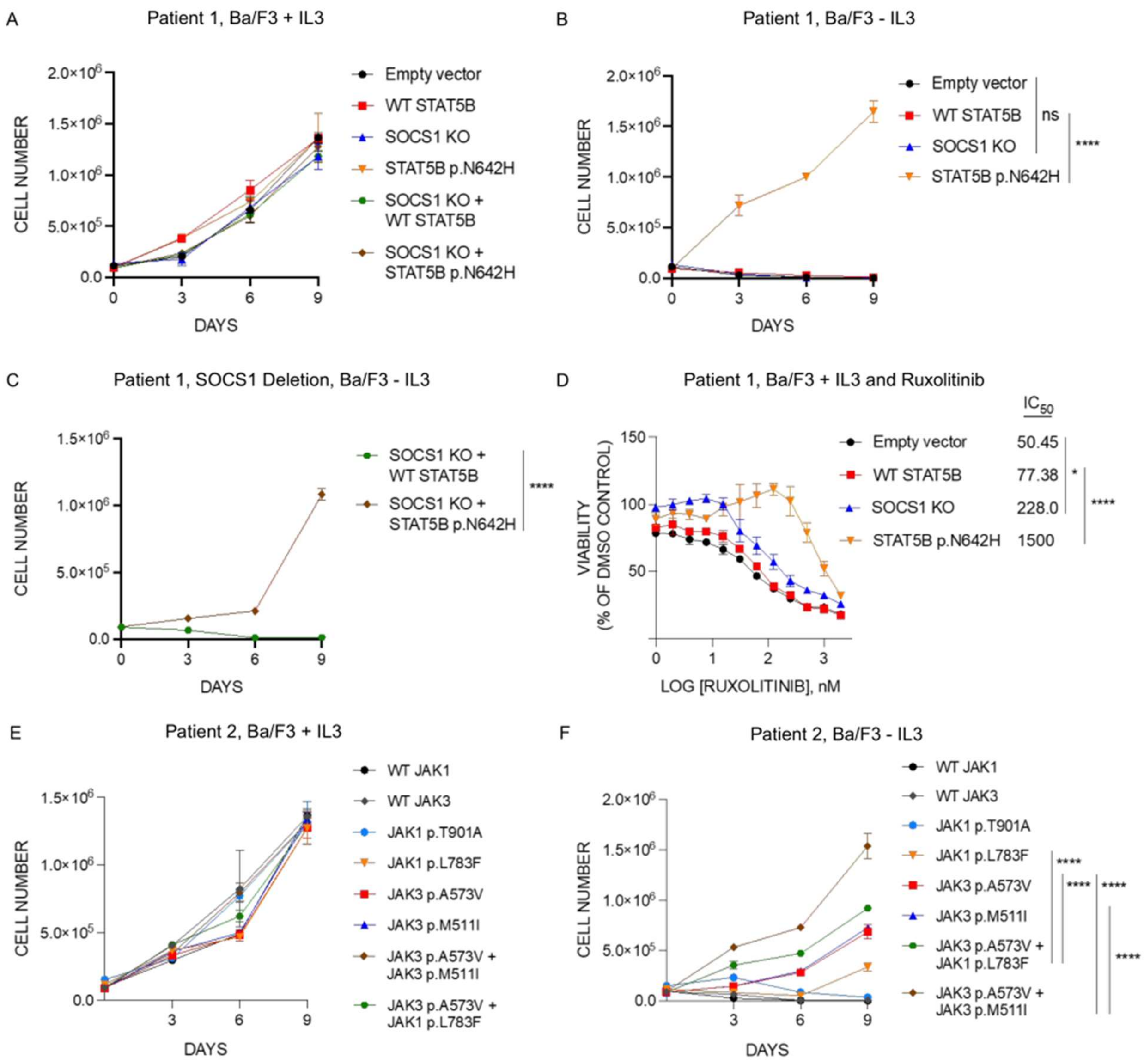

Supplemental Figure 4. Functional validation of mutations. (A-C) STAT5B N642H and SOCS1 KO expressing Ba/F3 IL-3 withdrawal. Retroviruses encoding lentiCRISPRv2-mcherry, wildtype STAT5B, lentiCRISPRv2-SOCS1, lentiCRISPRv2-SOCS1 with wildtype STAT5B, and lentiCRISPRv2-SOCS1 with STAT5B p. N642H were transduced into Ba/F3 cells. Transduced Ba/F3 cells were cell sorted based on GFP+ and/or mCherry+. On day 0, cells were seeded with **(A)** or without **(B,C)** IL-3 at 10^6 cells/ml. Results show cell counts and represent the means ± SD of triplicates. Statistical differences were tested using multiple comparison 2-way ANOVA. ns=not significant, ****p<0.0001. **(D)** Cell viability assay to determine IC_{50} values of Ruxolitinib in lentiCRISPRv2-mcherry, wildtype STAT5B, lentiCRISPRv2-

SOCS1, and STAT5B p. N642H with IL-3. 10^6 cells/ml were cultured with Ruxolitinib or vehicle control in a 96-well plate. Cell-titer Glo luminescent reagent (Promega) was added after 72 hours. IC50 value was determined using GraphPad Prism. Results show cell counts and represents the means ± SD of triplicates. Statistical differences were tested using multiple comparisons 2-way ANOVA.,*$p<0.01$,****$p<0.0001$. **(E-F)** JAK1 and JAK3 mutant Ba/F3 IL-3 withdrawal. Retroviruses encoding wildtype JAK1, wildtype JAK3, JAK3 p. A573V, JAK3 p. M511I, JAK1 p. L783F, JAK1 p. T901A, JAK1 p. L783F + JAK3 p. A573V, and JAK3 p. A573V + JAK3 p. M511I were transduced into Ba/F3 cells. Ba/F3 cells were sorted and cultured as in **A-C**. Created with BioRender.com.

References

1. Olsen EA, Whittaker S, Willemze R, Pinter-Brown L, Foss F, Geskin L, et al. Primary cutaneous lymphoma: recommendations for clinical trial design and staging update from the ISCL, USCLC, and EORTC. *Blood.* 2022;140(5):419-37.
2. Willemze R, Cerroni L, Kempf W, Berti E, Facchetti F, Swerdlow SH, et al. The 2018 update of the WHO-EORTC classification for primary cutaneous lymphomas. *Blood.* 2019;133(16):1703-14.
3. Bolotin DA, Poslavsky S, Davydov AN, Frenkel FE, Fanchi L, Zolotareva OI, et al. Antigen receptor repertoire profiling from RNA-seq data. *Nat Biotechnol.* 2017;35(10):908-11.
4. Beygi S, Duran GE, Fernandez-Pol S, Rook AH, Kim YH, and Khodadoust MS. Resistance to mogamulizumab is associated with loss of CCR4 in cutaneous T-cell lymphoma. *Blood.* 2022;139(26):3732-6.
5. Beygi S, Fernandez-Pol S, Duran G, Wang EB, Stehr H, Zehnder JL, et al. Pembrolizumab in mycosis fungoides with PD-L1 structural variants. *Blood Adv.* 2021;5(3):771-4.
6. Li H, and Durbin R. Fast and accurate short read alignment with Burrows-Wheeler transform. *Bioinformatics.* 2009;25(14):1754-60.
7. DePristo MA, Banks E, Poplin R, Garimella KV, Maguire JR, Hartl C, et al. A framework for variation discovery and genotyping using next-generation DNA sequencing data. *Nat Genet.* 2011;43(5):491-8.
8. McKenna A, Hanna M, Banks E, Sivachenko A, Cibulskis K, Kernytsky A, et al. The Genome Analysis Toolkit: a MapReduce framework for analyzing next-generation DNA sequencing data. *Genome Res.* 2010;20(9):1297-303.
9. Van der Auwera GA, Carneiro MO, Hartl C, Poplin R, Del Angel G, Levy-Moonshine A, et al. From FastQ data to high confidence variant calls: the Genome Analysis Toolkit best practices pipeline. *Curr Protoc Bioinformatics.* 2013;43(1110):11.0.1-.0.33.
10. Van der Auwera GA, and O'Connor BD. Sebastopol, CA: O'Reilly Media Sebastopol, CA; 2020.
11. Cibulskis K, Lawrence MS, Carter SL, Sivachenko A, Jaffe D, Sougnez C, et al. Sensitive detection of somatic point mutations in impure and heterogeneous cancer samples. *Nat Biotechnol.* 2013;31(3):213-9.
12. Diossy M, Sztupinszki Z, Krzystanek M, Borcsok J, Eklund AC, Csabai I, et al. Strand Orientation Bias Detector to determine the probability of FFPE sequencing artifacts. *Brief Bioinform.* 2021;22(6).
13. Choi J, Goh G, Walradt T, Hong BS, Bunick CG, Chen K, et al. Genomic landscape of cutaneous T cell lymphoma. *Nat Genet.* 2015;47(9):1011-9.
14. Daniels J, Doukas PG, Escala MEM, Ringbloom KG, Shih DJH, Yang J, et al. Cellular origins and genetic landscape of cutaneous gamma delta T cell lymphomas. *Nat Commun.* 2020;11(1):1806.
15. Goh G, Walradt T, Markarov V, Blom A, Riaz N, Doumani R, et al. Mutational landscape of MCPyV-positive and MCPyV-negative Merkel cell carcinomas with implications for immunotherapy. *Oncotarget.* 2016;7(3):3403-15.

16. Park J, Daniels J, Wartewig T, Ringbloom KG, Martinez-Escala ME, Choi S, et al. Integrated genomic analyses of cutaneous T-cell lymphomas reveal the molecular bases for disease heterogeneity. *Blood.* 2021;138(14):1225-36.
17. Park J, Yang J, Wenzel AT, Ramachandran A, Lee WJ, Daniels JC, et al. Genomic analysis of 220 CTCLs identifies a novel recurrent gain-of-function alteration in RLTPR (p.Q575E). *Blood.* 2017;130(12):1430-40.
18. Karczewski KJ, Francioli LC, Tiao G, Cummings BB, Alföldi J, Wang Q, et al. The mutational constraint spectrum quantified from variation in 141,456 humans. *Nature.* 2020;581(7809):434-43.
19. Zhang Y, Cheng K, and Choi J. TCR Pathway Mutations in Mature T Cell Lymphomas. *J Immunol.* 2023;211(10):1450-8.
20. Ou J, and Zhu LJ. trackViewer: a Bioconductor package for interactive and integrative visualization of multi-omics data. *Nat Methods.* 2019;16(6):453-4.
21. Van Loo P, Nordgard SH, Lingjærde OC, Russnes HG, Rye IH, Sun W, et al. Allele-specific copy number analysis of tumors. *Proc Natl Acad Sci U S A.* 2010;107(39):16910-5.
22. Gel B, and Serra E. karyoploteR: an R/Bioconductor package to plot customizable genomes displaying arbitrary data. *Bioinformatics.* 2017;33(19):3088-90.
23. Martin M. Cutadapt Removes Adapter Sequences From High-Throughput Sequencing Reads. *EMBnetjournal.* 2011;17(1):10-2.
24. Dobin A, Davis CA, Schlesinger F, Drenkow J, Zaleski C, Jha S, et al. STAR: ultrafast universal RNA-seq aligner. *Bioinformatics.* 2013;29(1):15-21.
25. Anders S, Pyl PT, and Huber W. HTSeq--a Python framework to work with high-throughput sequencing data. *Bioinformatics.* 2015;31(2):166-9.
26. Anders S, and Huber W. Differential expression analysis for sequence count data. *Genome Biol.* 2010;11(10):R106.
27. Huber W, von Heydebreck A, Sueltmann H, Poustka A, and Vingron M. Parameter estimation for the calibration and variance stabilization of microarray data. *Stat Appl Genet Mol Biol.* 2003;2:Article3.
28. Tieppo P, Papadopoulou M, Gatti D, McGovern N, Chan JKY, Gosselin F, et al. The human fetal thymus generates invariant effector γδ T cells. *J Exp Med.* 2020;217(3).
29. Love MI, Huber W, and Anders S. Moderated estimation of fold change and dispersion for RNA-seq data with DESeq2. *Genome Biol.* 2014;15(12):550.
30. Gu Z, Eils R, and Schlesner M. Complex heatmaps reveal patterns and correlations in multidimensional genomic data. *Bioinformatics.* 2016;32(18):2847-9.
31. Subramanian A, Tamayo P, Mootha VK, Mukherjee S, Ebert BL, Gillette MA, et al. Gene set enrichment analysis: a knowledge-based approach for interpreting genome-wide expression profiles. *Proc Natl Acad Sci U S A.* 2005;102(43):15545-50.
32. Mootha VK, Lindgren CM, Eriksson KF, Subramanian A, Sihag S, Lehar J, et al. PGC-1alpha-responsive genes involved in oxidative phosphorylation are coordinately downregulated in human diabetes. *Nat Genet.* 2003;34(3):267-73.
33. Liberzon A, Birger C, Thorvaldsdottir H, Ghandi M, Mesirov JP, and Tamayo P. The Molecular Signatures Database (MSigDB) hallmark gene set collection. *Cell Syst.* 2015;1(6):417-25.
34. Horwitz SM, Feldman TA, Hess BT, Khodadoust MS, Kim YH, Munoz J, et al. The Novel SYK/JAK Inhibitor Cerdulatinib Demonstrates Good Tolerability and Clinical

Response in a Phase 2a Study in Relapsed/Refractory Peripheral T-Cell Lymphoma and Cutaneous T-Cell Lymphoma. *Blood.* 2018;132(Supplement 1):1001-.
