# Supplementary material for: Addiction of primary cutaneous γδ T cell lymphomas to JAK/STAT signaling
Source: J Clin Invest. 2025 Apr 15;135(8):e180417. doi: 10.1172/JCI180417 (PMC11996904; doi:10.1172/JCI180417)
Supplement: Supplemental table 1 [file jci-135-180417-s152.pdf]

Patient 1 Clinical Presentation

Age	Sex	Stage	PET/CT	Clonotype	Prior Treatments
90y	F	$T_{3B}N_0M_0$	Negative for nodal or visceral disease	$V\delta1V\gamma5$	None

Patient 2 Clinical Presentation

Age	Sex	Stage	PET/CT	Clonotype	Prior Treatments
74y	F	$T_{2C}N_1M_0$	Affected lymph nodes in right groin. No visceral disease.	$V\delta1V\gamma5$	Pralatrexate<br>Brentuximab<br>Local Radiation
